# Impact of Orthodontic Treatment on Regenerative Endodontics in Immature Teeth: A Long-Term Case Study

**DOI:** 10.7759/cureus.46953

**Published:** 2023-10-13

**Authors:** Saeed Asgary, Bita Talebzadeh

**Affiliations:** 1 Endodontics, Iranian Centre for Endodontic Research, Research Institute for Dental Sciences, Shahid Beheshti University of Medical Sciences, Tehran, IRN; 2 Endodontics, School of Dentistry, Islamic Azad University of Medical Sciences, Tehran, IRN

**Keywords:** regenerative endodontics, calcium enriched mixture, orthodontics, dentistry, trauma

## Abstract

Regenerative endodontic procedures (REPs) are highly effective in treating immature teeth with pulpal necrosis. This case study aimed to determine the effects of orthodontic treatment on an immature necrotic tooth that had previously undergone REPs. The main objective was to explore the potential synergistic effects of REPs and orthodontic forces on root development. A 10-year-old patient with a previously traumatized and restored central incisor was treated using REPs. Initial resolution of symptoms and bony healing were observed. However, after three years, with the initiation of orthodontic treatment, there was a slight improvement in root length and thickness. This case underscores the potential positive interplay between orthodontic forces and REPs, warranting further in-depth studies.

## Introduction

Regenerative endodontic procedures (REPs) have emerged as a promising treatment option for necrotic immature teeth. This innovative approach is based on the principles of tissue regeneration, utilizing a combination of stem cells, chemical mediators, and bioactive scaffolds to restore the health of these compromised teeth. REPs offer benefits compared to apexification techniques, including the promotion of root development, thickening of dentinal root walls, and the achievement of apical closure. These advantages make REPs an appealing option for preserving the vitality and function of immature teeth that would otherwise require extraction [1].

Notwithstanding the efficacy of REPs in treating necrotic immature teeth, an evident knowledge gap persists regarding the interplay between orthodontic forces and teeth treated with REPs, and bridging this gap is of paramount importance. A comprehensive understanding of this synergy could unveil novel avenues for dental treatments, thus enhancing clinical outcomes. In a world where orthodontic interventions exert mechanical forces on teeth, mature vital teeth exposed to these forces sometimes face the brunt in the form of root resorption. Yet, the question lingers: "how do immature necrotic teeth, post-REPs, respond to orthodontic forces?"

By long-term evaluation of the current case, this study aspires to illuminate this knowledge gap, offering insights into the impact of controlled orthodontic forces on the efficacy of REPs.

## Case presentation

Patient presentation and diagnosis

A 10-year-old female patient reported to the Mehr Dental Clinic, Tehran, Iran, complaining of discomfort associated with her previously traumatized maxillary left central incisor (tooth #21). The injury to the tooth transpired one year before this presentation, and a subsequent restoration was performed. The adjacent tooth, #11, also bore evidence of the prior restoration.

**Figure 1:**
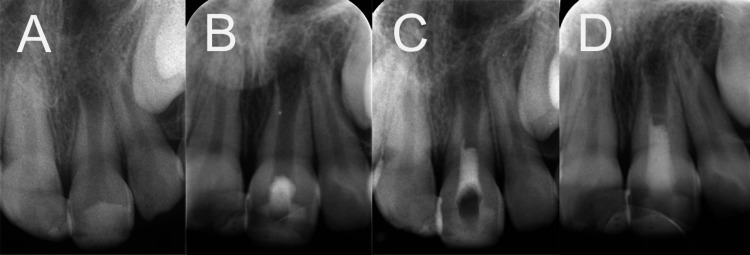
Radiographic progression of the immature necrotic tooth undergoing REPs (A) Initial presentation of the tooth showing the trauma history managed with composite resin restoration. (B) A radiograph following the commencement of REPs, showcasing the endodontic access cavity preparation, placement of a triple antibiotic paste, and temporary sealing. (C) Completion of REPs with coronal filling. (D) Post-coronal filling radiograph. REP, regenerative endodontic procedure

A thorough clinical examination entailed visual inspection, percussion tests, palpation, and pulp sensibility tests. Radiographic findings showcased an open apex in tooth #21, coupled with an associated apical lesion (Figure 1A). Based on these examinations, a diagnosis of pulp necrosis and symptomatic apical periodontitis was reached. Following in-depth consultations with the patient's parents and securing informed consent, it was resolved to administer REPs.

Regenerative endodontic procedure

**Figure 2:**
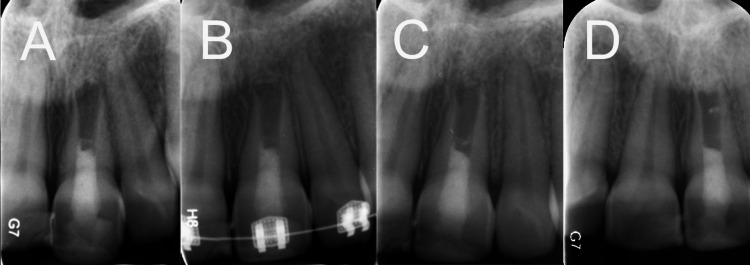
Radiographic follow-ups of the REP-treated tooth undergoing orthodontic treatment (A) A two-year follow-up radiograph revealing symptom resolution and signs of bony healing under the calcium-enriched mixture (CEM) cement, although root development remained limited. (B) Three years post-REPs, orthodontic intervention was introduced, with the specifics detailed in the report. (C) A follow-up radiograph one year after the initiation of orthodontic treatment, demonstrating a discernible increase in the root length and thickness. (D) A three-year follow-up radiograph indicating a normal periodontal ligament, a slight improvement in the root wall thickness and length. REP, regenerative endodontic procedure

Adhering strictly to established protocols, the REP was meticulously undertaken. This comprised access cavity preparation, rigorous chemomechanical cleaning of the infected canal, and the introduction of a triple antibiotic paste (a concoction of metronidazole, ciprofloxacin, and minocycline) during the inaugural appointment (Figures 1B-1D). This specific antibiotic blend was chosen, underpinned by its well-documented efficacy in analogous cases [2]. At the succeeding appointment, following canal cleansing and drying, bleeding was induced via overinstrumentation. The resultant blood clot was subsequently overlaid completely with an endodontic biomaterial, calcium-enriched mixture (CEM) cement. Notably, a two-year assessment post-REPs revealed abated symptoms, albeit with halted root development (Figure 2A).

Orthodontic procedures and patient's response

Subsequent to the consensus to embark on orthodontic treatment, fixed orthodontic appliances were employed. The maxillary left central incisor was subjected to bodily movement, leveraging an approximate force of 100 grams. Throughout the orthodontic regimen, the patient experienced minimal discomfort, and regular follow-ups discerned no noteworthy complications or adverse outcomes. Radiographs from the three-year follow-up demonstrated discernible albeit slight root maturation (Figures 2B-2D).

## Discussion

The case presented here furnishes a unique exploration of the synergistic application of regenerative endodontic procedures and orthodontic treatments in a traumatized maxillary central incisor with an open apex and pulp necrosis. Such a combination elucidates the conceivable influence of controlled orthodontic forces on root development in open-apex, necrotic teeth post-REPs.

As established, the primary objective of REPs encompasses the alleviation of symptoms and attainment of bony healing, a goal that was duly met within the initial three-year post-treatment interval [3]. Conversely, the secondary aim, concerning the augmentation of the root canal wall's thickness and elongation of root length, remained elusive.

The propensity for ongoing root development in REP-treated immature teeth is attributed to the viability of stem cells housed in the apical papilla. These cells, when stimulated by growth factors such as proteins from dentinal root walls and surrounding matrices, are poised to migrate into the root canal, fostering proliferation and differentiation [4]. Moreover, scaffolding agents like blood clots or fibrin amplify stem cell growth [5]. Yet, in scenarios characterized by protracted bacterial intrusion in immature teeth, as depicted in our case, the vitality of residual stem cells in the apical papilla and Hertwig's epithelial root sheath (HERS) could be jeopardized. Consequently, these cells might be ill-equipped to champion further root maturation.

Instituting orthodontic procedures three years post-REPs induced slight structural transformations in the immature root. Orchestrated orthodontic forces seemed to enhance vascular activity in the pulp and periapical domains, potentially activating stem cells in the apical papilla or HERS. Such activities may cause enhancements in root length, thickness, and the morphological transition of root walls. However, it is imperative to accentuate the study's limitations. As a singular case report, it does not offer irrefutable evidence linking orthodontic forces with optimized REP outcomes. A more expansive study encompassing larger cohorts and rigorous controls is required to substantiate our assertions. Still, these revelations underscore the potential merit of amalgamating REPs with orthodontics, thereby refining treatment paradigms and elevating patient outcomes.

## Conclusions

This illustrative case affords an initial glimpse into the potentially favorable interplay between orthodontic forces and REPs. It underscores the exigency for more robust, systematic investigations to validate these preliminary observations. From a clinical standpoint, these findings intimate that a synergistic approach could potentially optimize outcomes in analogous dental predicaments.
